# Charging and Aggregation of Nano-Clay Na-Montmorillonite in the Presence of Ciprofloxacin

**DOI:** 10.3390/nano15050389

**Published:** 2025-03-03

**Authors:** Chuanzi Zeng, Motoyoshi Kobayashi

**Affiliations:** 1 Graduate School of Science and Technology, University of Tsukuba, Tsukuba 305-8572, Ibaraki, Japan; 2 Institute of Life and Environmental Sciences, University of Tsukuba, Tsukuba 305-8572, Ibaraki, Japan

**Keywords:** clay, dynamic light scattering, electrophoresis, antibiotic

## Abstract

The transport and fate of antibiotics are significantly influenced by co-existing colloidal and nanosized substances, such as clay particles. Montmorillonite, a common clay mineral with a thin nano-sheet-like structure, enhances antibiotic (e.g., ciprofloxacin) mobility due to its strong adsorption properties. Nevertheless, little is known about how ciprofloxacin systematically influences the charging and aggregation properties of montmorillonite. This study examines the effect of ciprofloxacin on the electrophoretic mobility and hydrodynamic diameter of Na-montmorillonite under varying pH levels and NaCl concentrations. Results show ciprofloxacin promotes aggregation and alters the surface net charge of Na-montmorillonite at acidic to neutral pH, where ciprofloxacin is positively charged. At higher pH levels, where ciprofloxacin is negatively charged, no significant effects are observed. The observed aggregation behaviors align with predictions based on the Derjaguin–Landau–Verwey–Overbeek (DLVO) theory. Specifically, the slow aggregation regime, the fast aggregation regime, and the critical coagulation concentration are identified. The relationship between critical coagulation ionic strength and electrokinetic surface charge density is well explained by the DLVO theory with the Debye–Hückel approximations. Additionally, non-DLVO interactions are inferred. At low NaCl and ciprofloxacin concentrations, minimal changes in aggregation and surface charge suggest dispersed montmorillonite may facilitate ciprofloxacin transport, raising environmental concerns.

## 1. Introduction

With the development of industry, all kinds of environmental pollutions appear in our life. As modern science and technology progress, antibiotics are widely used in human, plant, and animal medical supplies and the growth promotion [1,2,3]. In many regions worldwide, the active compounds of antibiotics are discharged into the aquatic environment via municipal sewer systems without any special treatment [4]. While some antibiotics are readily degraded, others, such as fluoroquinolones, are more long-lasting and therefore remain in the environment for a longer period of time, spread farther, and can accumulate to higher concentrations [5]. Typically, antibiotics flow from wastewater into the surface water and eventually reach the soil [6,7,8]. In soil, antibiotics are often retained due to their strong adsorption onto clay particles, while also promoting the survival and proliferation of resistant bacteria, thereby accelerating their spread and affecting soil microorganisms in processes such as organic matter degradation, nitrogen cycling, and carbon cycling, ultimately posing potential threats to agricultural ecosystems and human health [2,9,10,11]. Studies have shown that antibiotic resistance genes originate from environmental bacteria, so variations in natural ecosystems may affect antibiotic resistance and thus human health even at the low doses [12,13]. Therefore, it is essential to develop strategies for the treatment and control of antibiotics in the environment.

**Figure 1:**
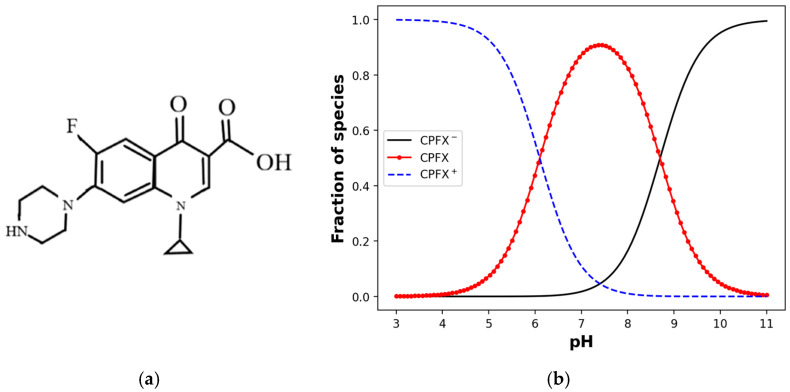
(**a**) Molecular structure and (**b**) speciation of ciprofloxacin (CPFX) under different pH conditions.

Fluoroquinolones have been used in human treatment, veterinary therapy, and agricultural production due to their wide range of activity and effective improvement of drug activation compared to other compounds. During this time, these antibiotics have increasingly entered the environment [14]. A fluoroquinolone antibiotic, ciprofloxacin (CPFX), is an organic ion widely used for bacterial infections and thus remains in the natural environment [15,16,17,18,19]. CPFX bears amphoteric charge and has strong sorption ability in soil systems so that CPFX can undergo different adsorption reactions to clays under acidic or alkaline conditions. Some research found that CPFX significantly changed the ζ-values of the colloids, especially bringing some colloids (e.g., hematite and kaolinite) close to the isoelectric point in alkaline environments, thus promoting colloid aggregation [20]. The reason is that CPFX being present as a cationic form CPFX^+^, due to amine group in the piperazine group, predominates when the pH of the solution is below 6.1. The net neutral state exists in neutral pH due to simultaneous protonation and deprotonation of amine and carboxylic acid groups [21], and an anionic state appears in an alkaline solution due to the carboxylic acid group [22]. Figure 1 illustrates the molecular structure and speciation of CPFX under varying pH conditions [23,24]. These shifts in charge state significantly influence CPFX’s electrostatic interactions and adsorption behaviors. Cationic antibiotics tend to bind to colloids and form stable aggregates that may lead to proximity transport and deposition. Anionic antibiotics tend to enhance colloidal dispersion and support their long-range transport [20].

**Figure 2:**
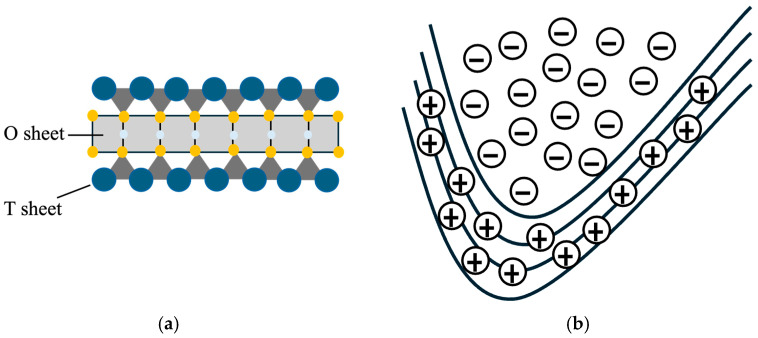
The structure of montmorillonite is composed of one O (octahedral) sheet between two T (tetrahedral) sheets (the blue circles are oxygen, white circles are silicon, and yellow circles are Al, Si) as shown in (**a**). The charge characteristics of montmorillonite are shown in (**b**).

Due to its large specific surface area and the permanent negative charge on its surface layer, montmorillonite (MMT) has a function as an effective adsorbent for pollutants such as heavy metals and organic contaminants in water and soil [25]. MMT is a 2:1 type clay mineral, featuring a structure similar to a sandwich, with one octahedral sheet positioned between two tetrahedral sheets (Figure 2a) [26,27,28]. The two silica tetrahedral sheets are positioned on the outside of the clay sheet, and the alumina octahedral sheet is in between the two silica sheets [29]. MMT particles have an irregular thin disc-like shape with larger diameter from 0.1 μm to 2 μm and 1 nm in thickness, and the average hydrodynamic diameter is around 0.5 μm [23]. The basal surface of MMT particle keeps the permanent negative charge, and the edges have a pH-dependent charge (Figure 2b) [30,31]. When the pH is lower, the edge surface shows the positive charge. At a higher pH, the edge surfaces become negatively charged. With the screening of the surface charge by increasing the concentration of electrolytes, MMT particles tend to attract each other in face-to-face or edge-to-face aggregation [32,33,34,35,36]. In summary, the strong cation exchange capacity of MMT is often used to treat contaminants in water and soil [37,38,39].

The transport of CPFX in soil is significantly influenced by its adsorption onto clay minerals [40,41,42], as colloidal and clay particles possess a high specific surface area that affects contaminant transport in both soil and water environments. The MMT has a higher adsorption ability than kaolinite and can remove the CPFX from sand media and facilitate the migration of CPFX [32,37,43,44]. The adsorption capacity of CPFX on MMT is affected by the pH and type of exchangeable cation [23]. The adsorption capacity of MMT for CPFX remained essentially unchanged between pH 3 and 8 but decreased significantly at pH > 8.7 [45]. MMT has a greater affinity for the cationic form of CPFX^+^ than for its CPFX^−^, as shown by the batch adsorption tests. The type of interlayer cations present influences MMT’s adsorption capacity. Notably, Na-MMT exhibits a higher adsorption capacity than Ca-MMT or Al-MMT [23,46]. A large number of researches have proved the ability and mechanism of adsorption of CPFX on MMT [23]. Colloid transport in porous media is primarily governed by aggregation and surface charge properties [47], playing a crucial role in determining the fate of nanoparticles and adsorbed contaminants. Therefore, we investigate whether CPFX influences MMT aggregation.

The aggregation and charging of colloidal and nanosized particles also affect the fate and transport of such particles with and without adsorbing chemicals known as colloid-facilitated transport and thus are important phenomena contributing to a deeper understanding of the risks of pollutants to humans and ecosystems [48,49,50,51,52,53]. The aggregation and dispersion of colloidal and nanosized particles are discussed by the Derjaguin–Landau and Verwey–Overbeek (DLVO) theory [54,55,56,57]. According to the DLVO theory, the stability of colloidal suspensions is governed by the interplay between van der Waals attractive forces and electric double-layer repulsive forces [56,58,59,60]. According to the DLVO theory, the colloidal particles are dispersed in the solution when the repulsive force is larger than the attractive force. With increasing the concentration of ions and/or decreasing the magnitude of surface electric potential, the attractive force will dominate, and thus the aggregation will occur. With increasing the electrolyte concentration, the aggregation rate of particles increases and reaches the maximum aggregation rate at the critical concentration called critical coagulation concentration (CCC) [58,61,62]. Beyond the CCC, the double-layer repulsive force disappears, causing particle destabilization. The CCC divides the aggregation into a fast aggregation regime and a slow aggregation regime and thus indicates the minimum concentration required for fast aggregation. Therefore, colloidal particles can be known whether the aggregation–dispersion follows the DLVO theory from the aggregation rate. So far, previous studies confirm that the aggregation and dispersion of many colloidal particles follows DLVO theory with and without organic ions [25,63]. As expected from the DLVO theory, MMT aggregates with increasing salinity [64].

The adsorption of CPFX on MMT may affect the charging and aggregation of MMT and alter the fate and transport of CPFX adsorbing MMT. In this regard, studying the charging and aggregation of MMT with CPFX is important. Nevertheless, systematic studies on how CPFX affects the charging and aggregation of MMT particles, particularly in terms of CCC with the DLVO theory, are scarce. In this context, we investigated the charging and aggregation of MMT in the presence of CPFX. The electrophoretic and dynamic light-scattering experiments were conducted to examine the charging and aggregation of MMT with and without CPFX under different salt concentrations and pH conditions. The aggregation behavior of MMT was analyzed using the DLVO theory, considering the effects of surface charge.

## 2. Materials and Methods

In the present study, the hydrodynamic diameters and electrophoretic mobility of montmorillonite (MMT) in the presence of ciprofloxacin (CPFX) were measured as a function of the concentration of NaCl and CPFX at different pHs. The measurements of the electrophoretic mobility and the hydrodynamic diameter were performed by using electrophoretic and dynamic light-scattering methods. The stability ratio, critical coagulation concentration (CCC), critical coagulation ionic strength (CCIS), and electrokinetic charge density from the zeta potential can be obtained from these experiments.

### 2.1. Materials

An aqueous Na-MMT dispersion was prepared from “Kunipia-F” powder (Kunimine Industry Co., Ltd., Tokyo, Japan), which was collected from the Tsukinuno mine station (Yamagata, Japan) and purified by the manufacturer. The further refining technique adopted from Tsujimoto et al. [65] was applied to the sample. Coarse components, such as silica sand in the material, were removed by sedimentation treatment. The clay particles were dispersed into a 2.0 M NaCl solution where the surface cations were replaced by sodium ions. Excess salt was removed by repeated dialysis against deionized water until the electric conductivity was reduced to 1.5 μS/cm. After these treatments, the samples were freeze-dried. In this experiment, the aqueous Na-MMT suspension with a concentration of 40 mg/L was prepared as a stock suspension.

Ciprofloxacin hydrochloride monohydrate (molecular mass: 367.8 g/mol) was purchased from the Tokyo Chemical Industry (Tokyo, Japan). The chemical formula of ciprofloxacin is C_17_H_18_FN_3_O_3_·HCl·H_2_O. NaCl solutions were used to control the salt concentration of the suspension. The concentrations of NaOH solution and HCl solution were used to control the pH. All of the MMT suspension and ciprofloxacin solution was kept in a fridge under shading before use to avoid degradation by UV light [1]. The CPFX solution was stored for less than one week before the experiment [19]. The suspension of MMT was sonicated to disperse before use. The examined pH values of suspensions were 3.98 ± 0.12, 6.04 ± 0.11, 9.98 ± 0.05 confirmed by a pH meter (HM-30R, TOA-DKK). All the deionized waters were prepared by the Elix 5 system (Merck Millipore, Tokyo, Japan).

### 2.2. Electrophoretic Mobility

When charged colloidal and nanosized particles are dispersed in the electrolyte solution under an external electric field, the charged particles migrate in a positive or negative direction depending on the sign of surface electric potential. Electrophoretic light scattering has been widely used to characterize the charging behaviors of colloidal and nanosized particles [66]. To examine the effect of the adsorption of CPFX on the charging of Na-MMT, the electrophoretic mobility of the MMT in the absence and presence of CPFX was measured. In this experiment, the concentration of Na-MMT was adjusted to 2 mg/L. The electrophoretic mobility of MMT at various concentrations of NaCl and CPFX under different pH conditions was measured by Zetasizer Nano (Malvern Panalytical Ltd., Malvern, UK). The electrophoretic mobility of MMT at each condition was recorded 3 times, and the average values were calculated.

The electrophoretic mobility *μ* is given by Equation (1):

(1) $$\mu =\frac{v}{E}$$

where *v* is the migration velocity under the applied electric field strength *E*. The Smoluchowski equation, Equation (2), is widely used to calculate the zeta potential from electrophoretic mobility:

(2) $$\mu =\frac{\varepsilon_{r}\varepsilon_{0}\xi }{\eta }$$

where *ε_r_* is the relative dielectric constant of the dispersion medium, *ε*_0_ is the dielectric constant of free space, *η* is dynamic viscosity of the suspension medium, and *ζ* is zeta potential. The surface charge density *σ* can be calculated by the Debye–Hückel (DH) approximation as following Equation (3):

(3) $$\sigma =\varepsilon_{r}\varepsilon_{0}\kappa \psi_{0}$$

where $\psi_{0}$ is the surface electric potential and is replaced by the zeta potential in this study, and thus the evaluated surface charge density is the so-called electrokinetic surface charge density throughout this study. κ is given by the following Equation (4):

(4) $$\kappa^{-1}={\left(\frac{\varepsilon_{r}\varepsilon_{0}k_{B}T}{2N_{A}q^{2}I}\right)}^{\frac{1}{2}}$$

$\kappa^{-1}$ is called the Debye length, which is a measure of the thickness of the diffuse double layer, *I* is ionic strength, *N_A_* is the Avogadro constant, *q* is the elementary charge, *k_B_* is the Boltzmann constant, and *T* is the absolute temperature.

### 2.3. Dynamic Lighting Scattering

Colloidal particles in suspensions are moved by the Brownian motion, which induces the collision between the particles [35]. Dynamic light scattering has been used to measure particle size in a solution or suspension [67]. The Brownian motion of the particles suspended in the solution causes fluctuations in the intensity of the scattered light [68]. The diffusion coefficient of particles *D* can be obtained by analyzing the fluctuation of light intensity, and the particle hydrodynamic diameter dh can be obtained from the Stokes–Einstein equation:

(5) $$d_{h}=\frac{k_{B}T}{3\pi \eta D}$$

The dynamic light-scattering method has been widely used to obtain the temporal variation in average hydrodynamic diameter (*d_h_*) of particles during aggregation [69].

The aggregation experiment was carried out at a concentration of Na-MMT of 2 mg/L as a function of the concentrations of NaCl and CPFX at different pHs of 4, 6, and 10. To simply confirm if the aggregation happens or not, the hydrodynamic diameter of MMT after 5 min from the sample preparation was measured. Here, the increase in hydrodynamic diameter was judged as a symptom of aggregation. To further investigate the detail process of aggregation, the temporal increase in hydrodynamic diameter due to aggregation was measured. In each set of experiments, the hydrodynamic diameter was measured up to 200 times, and the measurements were stopped when the value of dh reached more than 1000 nm. The data from the experiment showed the change in the hydrodynamic diameter of the particles with time (d*d_h_*/d*t*), indicating the aggregation rate. The stability ratio *W* is given by

(6) $$W=\frac{k^{f}}{k}=\frac{{{\left(dd_{h}/dt\right)}_{t\to 0}}^{f}}{{\left(dd_{h}/dt\right)}_{t\to 0}}$$

where *k^f^* and *k* refer to the fast and slow aggregation rates [70]. The initial slope of the temporal change in hydrodynamic diameter (d*d_h_*/d*t*)*_t_*_→0_ was used as a measure of the aggregation rate. The average value of (d*d_h_*/d*t*)*_t_*_→0_ at a high NaCl concentration was adapted as ${{\left(dd_{h}/dt\right)}_{t\to 0}}^{f}$.

The slow aggregation rate and fast aggregation rate can be distinguished by the critical coagulation concentration (CCC) from the stability ratio [71]. To obtain the CCC value from the experiment data of the stability ratio, Equation (7) can be used:

(7) $$\frac{1}{W}=\frac{1}{1+{\left(\frac{CCC}{C_{s}}\right)}^{\beta }}$$

where *Cs* is the salt concentration, and *β* is the slope of dlog (1*/W*)/dlog(*Cs*) in the slow aggregation regime [72].

From the CCC, the critical coagulation ionic strength (*CCIS*) could be calculated by

(8) $$CCIS=\frac{1}{2}\sum_{i}z_{i}^{2}C_{i}$$

where *z_i_* is the valence of *i*-th ion, and *C_i_* is the concentration of *i*-th ion at CCC [73].

## 3. Results and Discussion

### 3.1. The Electrophoretic Mobility and Hydrodynamic Diameter of Na-Montmorillonite

**Figure 3:**
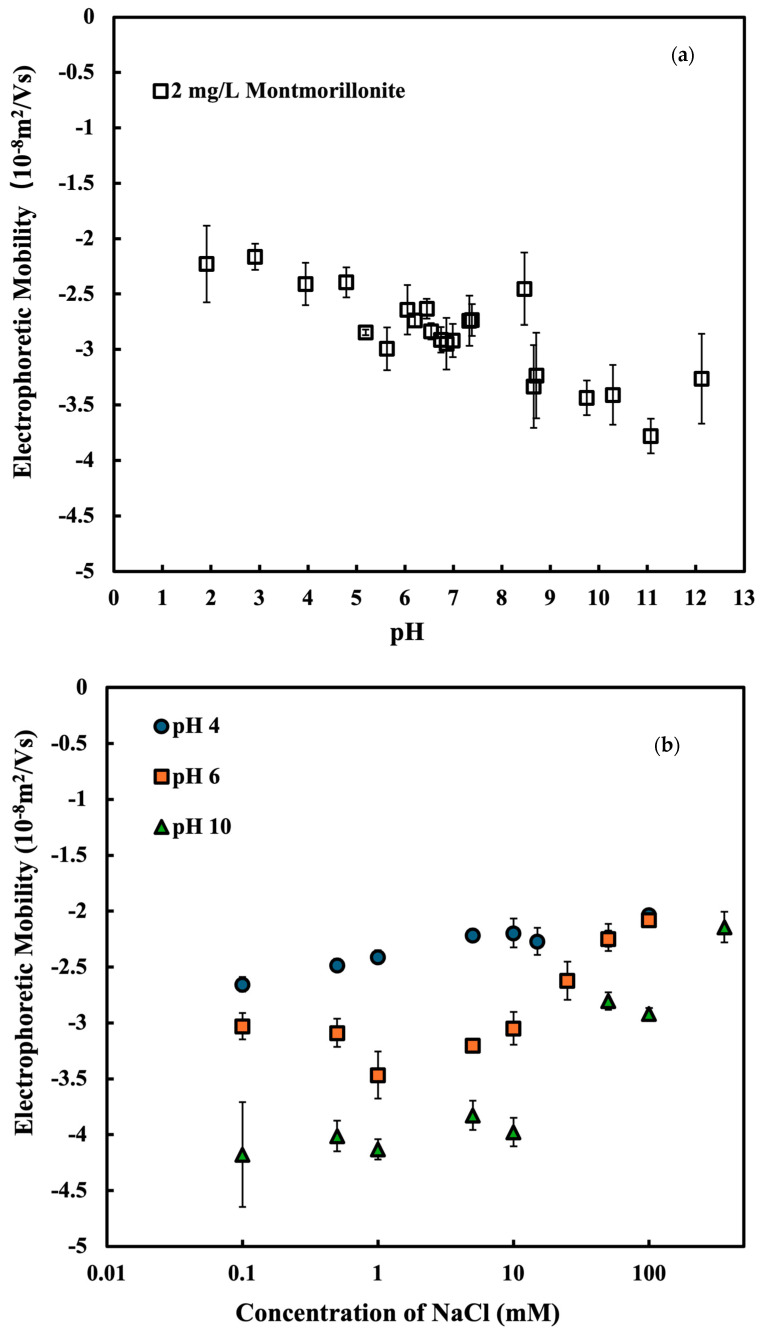
(**a**) Electrophoretic mobility of montmorillonite under different pH conditions with 10 mM NaCl solution. (**b**) Electrophoretic mobility of montmorillonite under different electrolyte concentrations at pH 4, 6, 10 (±0.1).

Figure 3 shows the electrophoretic mobility (EPM) of MMT as a function of the NaCl concentration. From Figure 3, we can see that, even at different pHs, the EPM of Na-MMT keeps a negative charge. Increasing the pH caused MMT’s negative EPM to rise; the isoelectric point (IEP) in the pH range does not exist. As pH increases, the absolute value of the EPM increases, indicating that the negative surface charge of montmorillonite (MMT) is enhanced. This may be due to the deprotonation of edge sites at a higher pH (Si-OH → Si-O^−^). This behavior is attributed to the structure of MMT, where the basal plane holds a constant negative surface charge due to its structural characteristics called isomorphous substitution [46,52]. Due to the interaction between a H+ ion or OH- ion and the edge of clay particles, the charge of the edge of clay particles can be positive, neutral, or negative [39]. At around pH 6, the positive-edge charges diminish, leaving the basal surface with a dominant negative charge even though the edge charge remains pH-dependent [46]. Figure 3b indicates that the EPM of MMT keeps a negative charge even as the NaCl concentration increases. Surface chemistry of the MMT could be the source of its intrinsic negative charge. That is, the negative charge on the MMT surface is not always neutralized by a rise in NaCl content coupled with an increase in the ionic strength of the solution.

**Figure 4:**
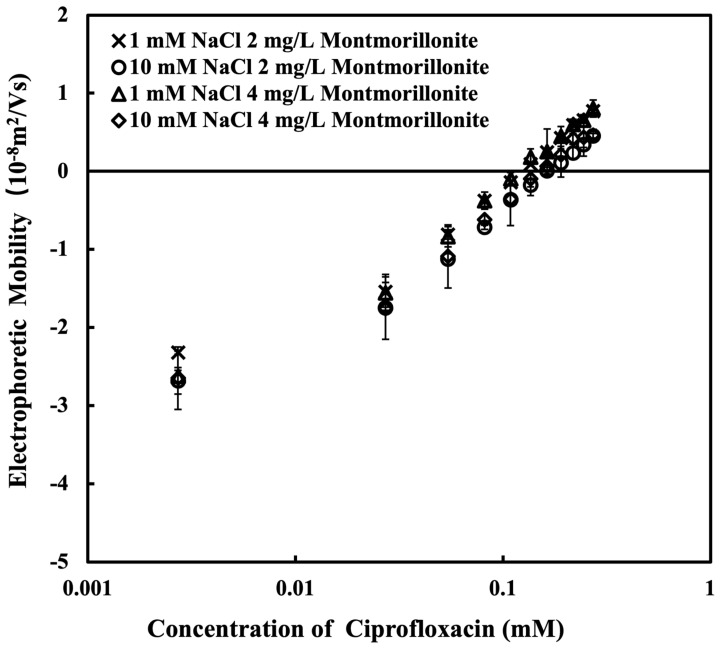
Electrophoretic mobility of montmorillonite with ciprofloxacin at different NaCl concentration and pH 4 after 5 min reaction.

Figure 4 shows the EPM of MMT in the presence of CPFX at 1 and 10 mM NaCl and a pH of 3.98 ± 0.12. As the concentration of ciprofloxacin is increased, the EPM of the MMT increases due to the adsorption of CPFX. The EPM of MMT is reversed from the negative to the positive. At the low CPFX concentration, the EPM shows the negative value because of the net negative-charge density on the surface of MMT. With increasing the concentration of CPFX, the cationic form of CPFX adsorbed onto the MMT induces the charge reversal [39]. The NaCl concentration does not significantly impact the EPM of MMT in the presence of CPFX. The charge reversal occurs at CPFX concentrations between 0.0815 mM and 0.1087 mM, defining the IEP, where the zeta potential reaches zero. Identifying the IEP provides insight into colloidal stability under different pH and electrolyte conditions.

**Figure 5:**
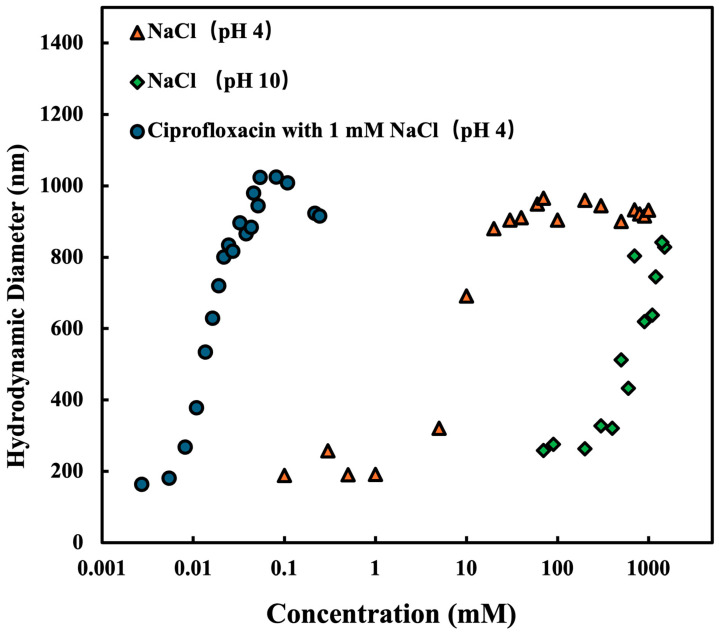
Hydrodynamic diameter of montmorillonite at 4 mg/L as a function of the NaCl concentration at pH 4 and 10 and the ciprofloxacin concentration at pH 4 with 1 mM NaCl solution. The data show the hydrodynamic diameter of the montmorillonite with ciprofloxacin immediately.

To verify if the addition of CPFX affects the aggregation of MMT, we measured the hydrodynamic diameter of MMT as a function of the concentration of CPFX and NaCl. The results are displayed in Figure 5. The hydrodynamic diameter of stable Na-MMT was around 170 nm to 200 nm. Similar to solutions containing only NaCl, the hydrodynamic diameter of Na-MMT rises with increasing the concentration of NaCl when CPFX is included. Furthermore, at both acidic and alkaline conditions, the size of MMT grew with the concentration of the salt solution, suggesting that pH has minimal impact on MMT aggregation induced by salt solutions. Nevertheless, in alkaline conditions, the salt solution had no effect on the EPM of MMT, suggesting that additional forces are involved in the aggregation of MMT in alkaline environments. Water molecules may have an altered configuration around the particles in alkaline settings, creating hydrophobic areas that promote particle aggregation.

**Figure 6:**
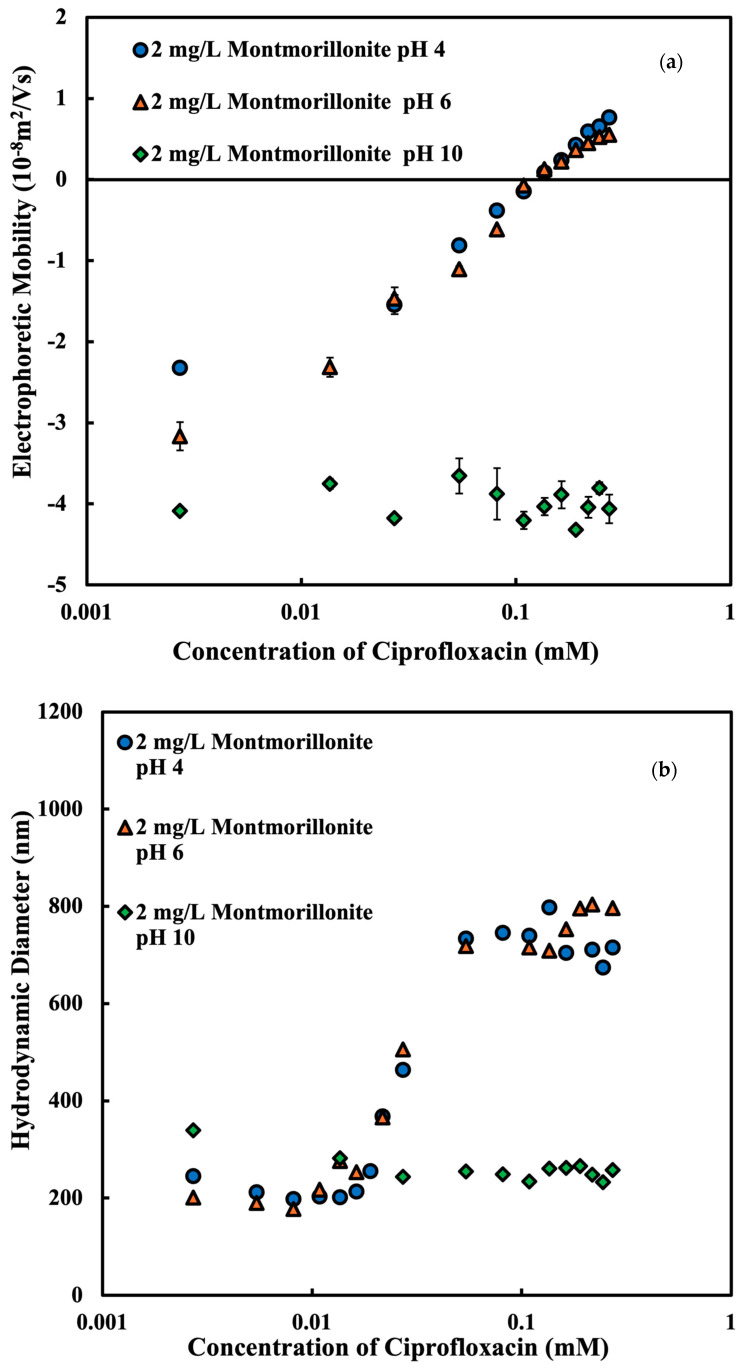
Electrophoretic mobility and hydrodynamic diameter of montmorillonite at 2 mg/L as a function of the ciprofloxacin concentration at pH 4.0, 6.0, and 10.0 with 1 mM NaCl solution. The data show electrophoretic mobility (**a**) and hydrodynamic diameter (**b**) of montmorillonite adsorbed with ciprofloxacin on.

In order to determine the effect of pH on the charging and aggregation behavior of MMT in the presences of CPFX, we measure the EPM and hydrodynamic diameter of MMT at 2 mg/L as a function of the CPFX concentration at pH 3.98 ± 0.12, 6.04 ± 0.11, 9.98 ± 0.05 with 1 mM NaCl solution, as shown in Figure 6. At a pH of 4 and 6, with increasing the concentration of CPFX, the hydrodynamic diameter of MMT increases, and the magnitude of EPM reduces. These trends on MMT can be explained as follows: the decrease in the absolute value of EPM is due to the adsorption of oppositely charged CPFX, and the reduced magnitude of EPM weakens the repulsive force between particles and increases the average size by aggregation [74]. In an alkaline condition, the EPM is constantly negative, showing the negative charge of MMT irrespective of CPFX concentration. Both CPFX and MMT under alkaline conditions have a negative charge that causes strong repulsion between them. The repulsion between CPFX and MMT at a high pH prevents the adsorption, and thus the EPM is more or less constant. Without the charge neutralization, the aggregation of MMT cannot happen at a low salt concentration. CPFX has hydrophobic regions, such as the fluoroquinolone core and aromatic rings, which may form hydrophobic patches on the MMT surface. This may enhance particle attraction through hydrophobic interactions, which explains why aggregation is stronger under acidic conditions, where CPFX is positively charged and adsorbs more easily, and weaker under alkaline conditions. CPFX has been demonstrated in earlier research to dramatically change the zeta potential of colloidal particles [20]. These conclusions are further supported by our findings, which demonstrate that the cationic form of CPFX^+^ influences the charge of Na-MMT through electrostatic interactions under acidic to neutral pH conditions, promoting aggregation.

### 3.2. The Stability Ratio of Na-Montmorillonite

**Figure 7:**
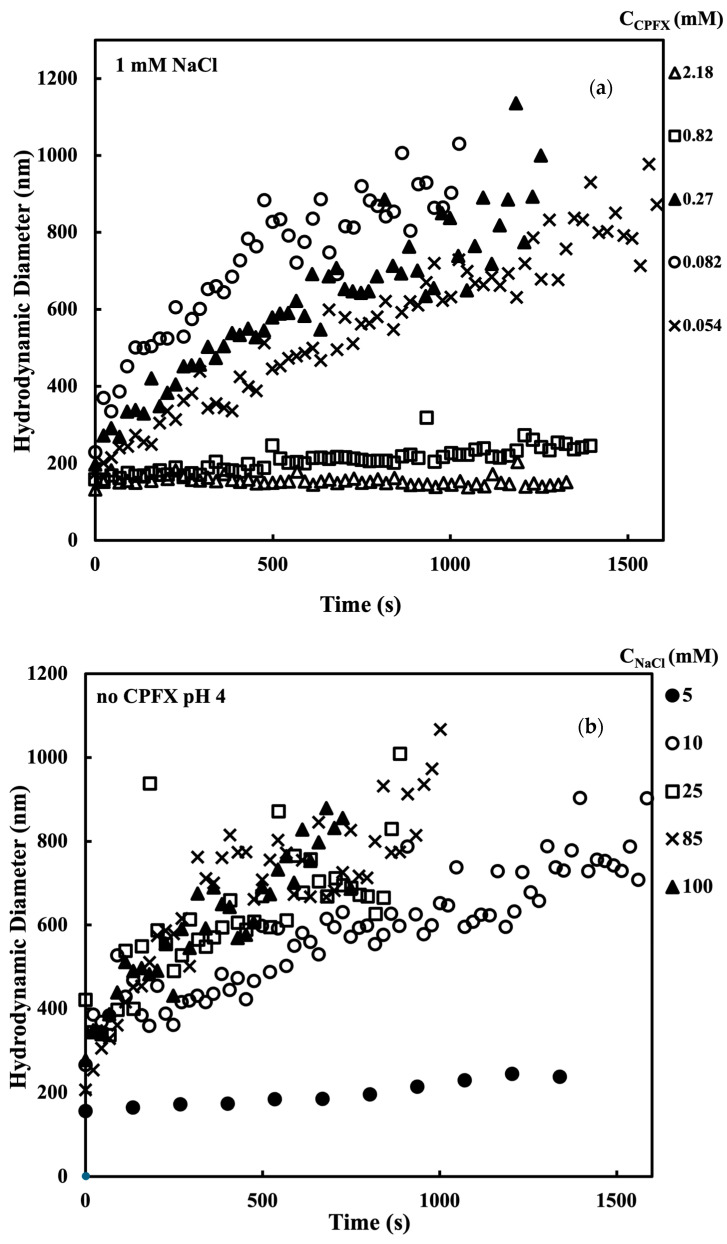

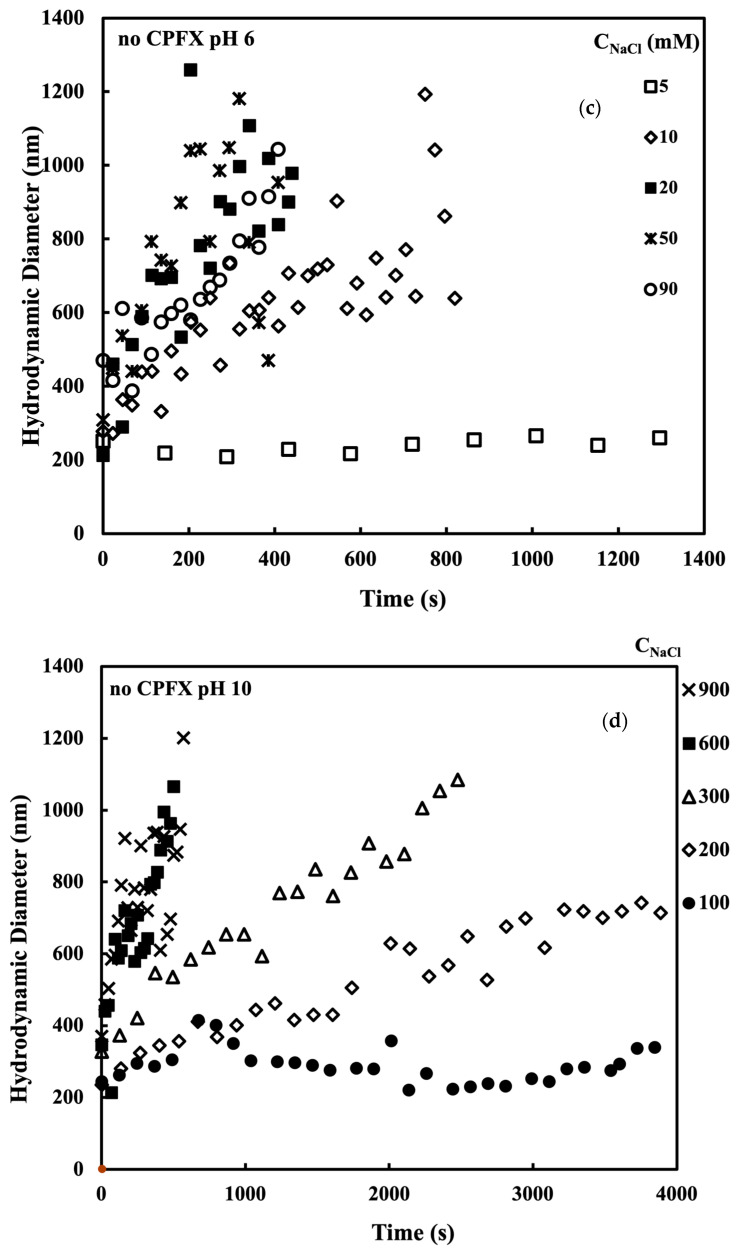
The temporal increase in hydrodynamic diameter of montmorillonite as a function of time at 2 mg/L at pH 4.0, 6.0, and 10.0. The hydrodynamic diameters of montmorillonite in the presence of ciprofloxacin at pH 4 with 1 mM NaCl are shown in (**a**), and the hydrodynamic diameters of montmorillonite with NaCl under pH 4, 6, and 10 are shown in (**b**–**d**).

The hydrodynamic diameter vs. time curves with various CPFX and salt concentrations are shown in Figure 7. As shown in Figure 7a, when the concentration of CPFX was higher than 0.109 mM, the hydrodynamic diameter of the MMT increases with time, resulting in the increased size over 1000 nm at certain conditions. The initial aggregation rate of MMT also increased with increasing the concentration of CPFX and then reached the maximum. However, when the concentration of CPFX is around 1.63 mM, the hydrodynamic diameter of MMT remains constant at 150 nm to 180 nm. The result showed that the higher CPFX concentration would interfere with the aggregation of MMT. In this study, the initial aggregation rate given in ($\frac{{dd}_{h}}{dt}$)_t→0_ is used to obtain the stability ratio W, as shown in Equation (6). This notation represents the derivative of dh with respect to t, evaluated as t approaches 0. The relationship between the hydrodynamic diameter and time was fitted with a third polynomial equation, and the coefficient of the first-order term of the equation was extracted to represent the initial slope ($\frac{{dd}_{h}}{dt}$)_t→0_. Figure 7b–d also shows a plot of the size vs. time at different NaCl concentrations without CPFX. The degree of increase in MMT size at different pH conditions appears to be related to the NaCl concentration, suggesting that higher ionic strength promotes aggregation [34]. A similar trend is seen at pH 4 and pH 6, where the hydrodynamic diameter of MMT increases with the increasing NaCl concentration. At pH 10, however, the increase in MMT size is more pronounced at higher concentrations (e.g., NaCl solution is 300 mM and above) compared to lower concentrations. Similar to the explanation for Figure 3, the higher magnitude of net surface charge of MMT at pH 10 likely contributes to increased electrostatic repulsion, making it necessary to require greater ionic strength for the screening of double-layer repulsion.

**Figure 8:**
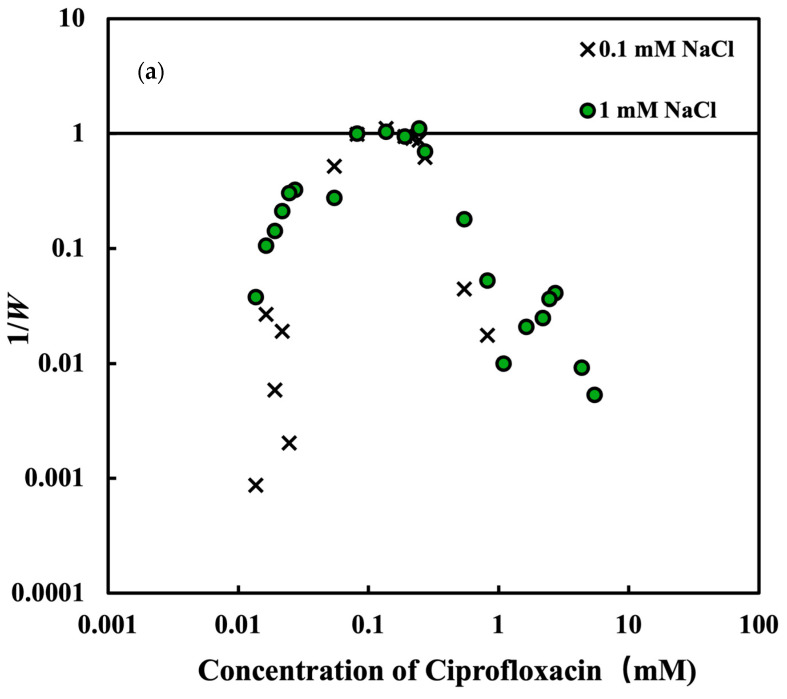

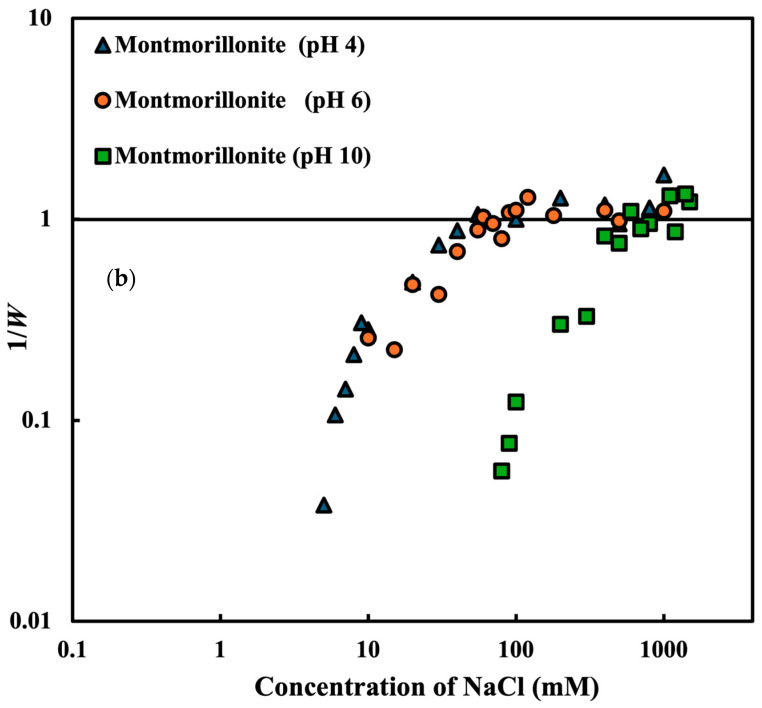
The data show the inverse stability ratio (1/*W*) of montmorillonite as a function of ciprofloxacin or NaCl at 2 mg/L concentration of montmorillonite at pH 4.0, 6.0, and 10.0. (**a**) montmorillonite with NaCl solution under different pH conditions; (**b**) montmorillonite with ciprofloxacin at 1 mM NaCl and pH 4 condition.

To look further into the aggregation mechanism of MMT in the presence and absence of CPFX, the inverse stability ratio 1/*W* of MMT vs. the concentrations of CPFX and NaCl concentration are shown in Figure 8. The different symbols represent different conditions. The inverse stability ratio 1/*W* of MMT without CPFX under different pH condition shows the increasing trends with increasing the concentration and reaches the constant over a critical concentration. The stability ratio W is defined as Equation (6), where ddhdtt→0f is the ddhdt in the fast aggregation regime attained at higher NaCl concentrations. The lowest electrolyte concentration necessary for the fast aggregation of colloidal particles in a suspension is known as the critical coagulation concentration (CCC). Generally, in the slow aggregation regime, the aggregation rate increases with increasing the electrolyte concentration, which indicates that the van der Waals attractive force gradually becomes dominant. With the increase in electrolyte concentration over the CCC, the aggregation rates become constant due to the screening of the electric double layer. In that condition, the aggregation rate of particles is independent of the salt concentration. In the presence of CPFX at a pH of 4, with increasing the CPFX concentration, the value of 1/*W* increases, reaches the maximum, and decreases as shown in Figure 8a. In this case, when the CPFX concentration is initially increased, CPFX can adsorb onto the MMT surface, partially neutralizing the surface charge and thus reducing electrostatic repulsion between particles, making the particles aggregate. The aggregation of MMT in the presence of CPFX under a pH 4 condition also displays the fast and slow aggregation regions of MMT. Compared to the salt induced coagulation, the CCC of CPFX is much smaller. According to the DLVO theory, a high NaCl concentration induces aggregation by the compression of the double layer. CPFX induces aggregation by the charge neutralization, as shown in Figure 4. As the concentration of CPFX increases, the repulsive force becomes weaker due to the charge neutralization. Consequently, MMT aggregates. With increasing the concentration of CPFX, the charge reversal is strong and the repulsive force becomes stronger, resulting in a slower aggregation rate. As a result, re-stabilization is observed at a high concentration of CPFX.

Compared to CPFX, the CCCs with NaCl were different under different pH conditions, as shown in Figure 8b. With increasing the pH, the CCC increases. The CCC is smaller when the pH is lower. This lower CCC at low pH is probably because the magnitude of the EPM of MMT is lower and induces aggregation more easily. In addition, the face-to-edge attraction might contribute to this lower CCC. In a near-neutral pH environment, the electrostatic repulsion between particles is greater than that in an acidic environment because the surface of MMT is more negatively charged. To overcome these repulsive forces and accomplish aggregation, a higher concentration of electrolytes (higher CCC) is needed. When the pH is 10, the edge and face of MMT show the negative charge, and the face-to-edge attraction is not expected [52]. In that condition, the aggregating montmorillonite needs more salt concentration to compress the electric double layer of the particles.

The CCC was extracted by using Equation (7) and is listed in Table 1. The calculated results are also consistent with those in Figure 8. CPFX significantly lowers the CCC compared to NaCl, indicating strong promotion of aggregation at pH 4. Higher pH increases CCC, indicating that MMT becomes more stable and resists aggregation as pH increases. It should be noted that this CCC of CPFX could be higher than an environmental concentration of CPFX and thus a stable complex of Na-MMT and CPFX would be expected in nature at a lower ionic strength.

### 3.3. The Relationship Between Critical Coagulation Ionic Strength and Surface Charge Density Based on the DLVO Theory

According to the DLVO theory, the interaction energy (*V*) between colloidal particles can be written as follows:

(9) $$V=V_{vdw}+V_{edl}$$

where the *V_vdW_* is van der Waals interaction potential energy, and *V_edl_* is the electric double-layer interaction potential energy. The van der Waals interaction energy for spheres is written as

(10) $$V_{vdw}=-\frac{Ha}{12h}$$

by using the Derjaguin approximation, where *H* is the Hamaker constant, *h* is the surface separation distance (or the distance from the surface), and *a* is the radius of the colloidal particle. With the Debye–Hückel (DH) approximation, by assuming the lower magnitude of surface potential, the double-layer interaction energy can be written as follows:

(11) $$V_{edl}=2\pi a\varepsilon_{0}\varepsilon_{r}\psi_{0}^{2}e^{-\kappa h}$$

*ψ*_0_ is the surface electric potential; κ is the inverse Debye length, which is related to the ionic strength of the electrolyte solution; $\varepsilon_{0}$ is a physical constant that describes how electric fields interact with the vacuum; and $\varepsilon_{r}$ indicates how much the electric field is reduced within a material compared to a vacuum. With the DH approximation, the surface charge density can be also expressed as Equation (3).

When the interaction energy satisfies *V* = 0 and d*V*/d*h* = 0 at a certain separation distance, the relationship between critical coagulation ionic strength (CCIS) and the surface charge density can be given by

(12) $$CCIS={\left(\frac{9}{8\pi e^{2}}\right)}^{\frac{1}{3}}\frac{1}{\lambda_{B}}{\left(\frac{\sigma^{2}}{\varepsilon_{0}\varepsilon_{r}H}\right)}^{\frac{2}{3}}$$

where the *λ_B_* is the Bjerrum length shown as Equation (13):

(13) $$\lambda_{B}=\frac{q^{2}}{4\pi k_{B}T\varepsilon_{r}\varepsilon_{0}}$$

Within the framework mentioned above, in contrast to the CCC, which is often discussed with the valence of ions, the CCIS only depends on the Hamaker constant and the surface charge density and not on ionic valence [75].

**Figure 9:**
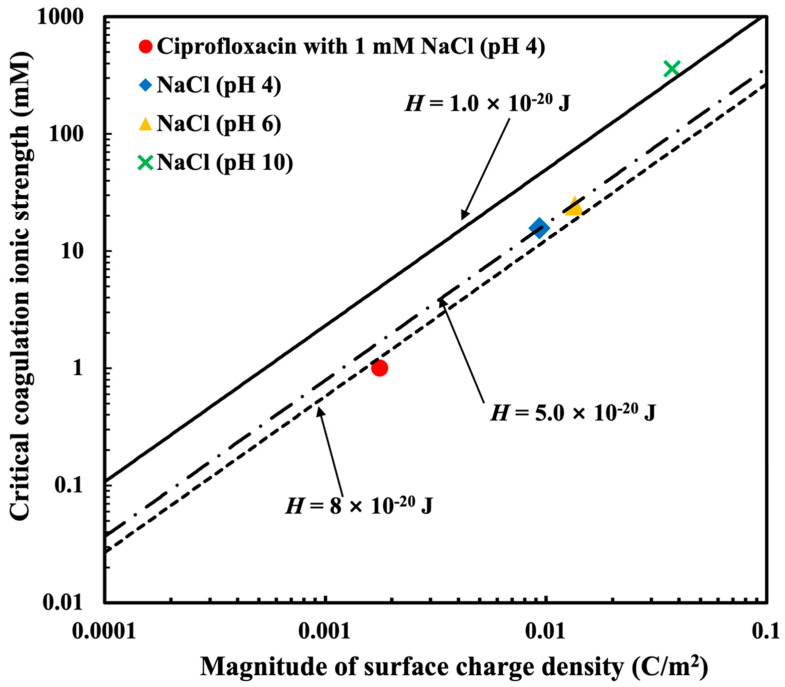
The critical coagulation ionic strength against surface charge density in the case of ciprofloxacin or NaCl solutions with 2 mg/L montmorillonite. The symbols are experimental data. The solid and dashed lines are the DLVO predictions using the Hamaker constant *H* of 1, 5, and 8 × 10^−20^ J for montmorillonite in water.

In this section, to discuss the applicability of the DLVO theory with DH approximation for the aggregation of MMT, we use the relationship between the critical coagulation ionic strength (CCIS) and surface charge density, as shown in Figure 9. In this figure, the symbols are the experimental data obtained from the aggregation of MMT, and the lines are the ones predicted by Equation (12) based on the DLVO theory with DH approximation. The surface potential of the MMT particles is assumed to be equal to the zeta potential, and therefore the experimental surface charge is the so-called electrokinetic one. The zeta potential around the CCIS is not so high. Thus, in this condition, we can reasonably use Equation (12).

**Figure 10:**
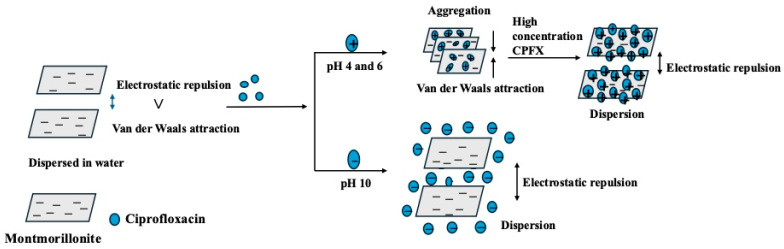
Aggregation of montmorillonite in the presence of ciprofloxacin.

In Figure 9, the red circle symbol is the data for the montmorillonite in the presence of ciprofloxacin with 1 mM NaCl under a pH 4 condition. Other symbols correspond to MMT without CPFX under pH 4, 6, and 10. The lines are the theoretical values calculated by Equation (12) with different values of the Hamaker constant of MMT in water around 5.0 × 10^−20^ J [76]. As shown in Figure 9, the experimental data and the theoretical ones reasonably agree well, and thus the relation can be rationally described by the DLVO theory. Figure 10 illustrates the aggregation of montmorillonite based on the DLVO theory. Previous research has determined that MMT exhibits a strong adsorption capacity for CPFX and significantly enhances its transport in saturated sand [39,45,77]. In this study, we further found that CPFX strongly influences the aggregation behavior of MMT. Figure 6b also shows that the presence of CPFX significantly affects MMT aggregation at lower pH levels, while at pH 10, the dispersion of MMT is maintained because of the electrostatic repulsion between MMT particles.

Particle aggregation is more likely to occur when the Hamaker constant is higher because of the increased attractive interaction. In contrast, when the Hamaker constant is smaller, the attraction is less, and the system tends to be more dispersed. From Figure 9, the CCIS of MMT with only NaCl under pH 10 is located on the line with the smaller Hamaker constant around 1.0 × 10^−20^ J, which indicates that the aggregation of MMT is harder under pH 10 conditions. When the pH is over 6.5, the Si-OH would be the dominant sites to make the edge change into a negative charge and the heterogeneity of the MMT layer to disappear [52]. That is, in alkaline conditions, the edge of MMT is negatively charged. Therefore, at pH 10, the edge–face attraction between the positively charged edge and negatively charged face can be ruled out. At pH 4 and 6 with only NaCl, the experimental CCIS is on the theoretical line with the higher Hamaker constant of 5.0 × 10^−20^ J, as shown in Figure 9. The positively charged edges of MMT exist below pH 6.5 due to the protonation of Al-OH sites on the edge. The edge–face attraction between the positively charged edge and the negatively charged face can provide additional attraction, which could be a reason for the higher Hamaker constant at pH 4 and 6 than that at pH 10. With CPFX at pH 4, the CCIS is near the theoretical line with around 8.0 × 10^−20^ J lager than the 5.0 × 10^−20^ J. It is possible that the adsorption of CPFX on the negatively charged face of MMT causes additional attractive interactions via charge-patch heterogeneity and hydrophobic interactions. CPFX molecules contain hydrophobic groups, which may form hydrophobic regions upon being adsorbed onto the MMT surface. Hydrophobic interactions promote particle aggregation, further contributing to non-DLVO interactions. Therefore, the aggregation of MMT induced by CPFX is not solely explained by the DLVO theory but also involves non-DLVO interactions. Moreover, this additional interaction could be the reason for the higher Hamaker constant of MMT in the presence of CPFX. The aggregation of MMT in the presence of CPFX at pH 4 is promoted more than the aggregation of MMT with only the NaCl solution because of this higher Hamaker constant and weaker electrokinetic surfaces’ charge density.

## 4. Conclusions

In this study, the aggregation and charging of montmorillonite (MMT) with and without ciprofloxacin (CPFX) were systematically studied by measuring the electrophoretic mobility and the hydrodynamic diameter. The adsorption of CPFX on Na-MMT significantly impacted the aggregation behavior of MMT, as indicated by the changes in the critical coagulation concentration (CCC) and critical coagulation ionic strength (CCIS). The high concentration of CPFX can change the net surface charge of MMT and induce the aggregation of MMT. In acidic and neutral pH solutions, the CPFX concentration influenced the aggregation of Na-MMT, and the Derjaguin–Landau and Verwey–Overbeek (DLVO) theory effectively explained this behavior. In contrast, at a high pH, where CPFX carries a negative charge, its adsorption on Na-MMT was inhibited, resulting in minimal impact on aggregation and charging. At lower concentrations of NaCl and CPFX, the change in aggregation and charging of MMT is weak. Consequently, CPFX forms stable colloid-pollutant complexes with dispersed MMT in natural aquatic environments because the environmental concentration of CPFX is generally much lower than the CCC found in our experiment. These complexes may enhance CPFX mobility in aquatic environments by reducing its retention in soil or on sediment, potentially posing environmental risks. The findings of this study can help predict the migration of antibiotics across environmental media, providing a scientific basis for soil remediation, water treatment, and antibiotic pollution-management strategies.

## Figures and Tables

**Table 1:** Critical coagulation concentration (CCC) of montmorillonite with or without ciprofloxacin.

	Ciprofloxacin (pH 4)	NaCl (pH 4)	NaCl (pH 6)	NaCl (pH 10)
**CCC (mM)**	0.034	15.69	24.54	360.19

## Data Availability

The data are available from the authors upon reasonable request.
